# Keystone Veterinary Medical Association

**Published:** 1894-02

**Authors:** Walter L. Hart

**Affiliations:** Secretary


The minutes of the previous meeting were read and approved.

*Secretary.*

New York, January 9th, 1894.

### KEYSTONE VETERINARY MEDICAL ASSOCIATION.

The regular meeting of the Keystone Veterinary Medical Association was held Tuesday evening, January 9th. The meeting was called to order by *Dr. Charles Lintz*, who was called to the chair in the absence of the president, Dr. Charles T. Gœntner. On roll call Drs. Rhoads, Lintz, Hoskins, Bridge and W. L. Hart answered. The minutes of the previous meeting were read and approved.

The report of the Board of Censors was next made. The following resolutions, after some discussion, were adopted by the association, and the secretary directed to transmit them to the respective persons to whom they were addressed:

Philadelphia, Pa., January 9th, 1894.

HON. WM MANN,

Prothonotary of the County of Philadelphia:—

DEAR SIR:—We, the officers and members of the Keystone Veterinary Medical Association, respectfully call your attention to the many changes that have occurred from death, removal, etc., in connection with the veterinary medical registry of this county, and that the law under which said registries were made enumerates as one of its features that a record shall be kept of such changes, and a suitable minute made upon the registry book.

Appreciating your many kind services to us in the past in this direction, we would respectfully request that a record be made in conjunction with the following names as those who have died since said registration was made, namely, Drs. Wm. H. Hitchman, George Kilbride, James McCourt, John Lovett, John B. Berry, Theodore Apeldorn, L. C. Campbell.

As removals from the city, Drs. R. S. Huidekoper, who is a resident of New York City; Wm. A. Birch, N. A. Cohen, Antoni Maurice, and Edward McCarty, now residents of the State of New Jersey; Chas. Lintz, now a resident of Chester, Delaware County; J. P. Zuill, now a resident of Delaware; Harry Walter, who has removed to Wilkesbarre, Luzerne County, and Dr. L. O. Lusson, who has removed to Ardmore, Montgomery County.

We would further request that you, personally, examine the veterinary medical registry, and give necessary directions as you may deem proper, inasmuch as we have found by examination that of many of these registries their addresses have never been placed with them, and in some one or two instances the name of the person registering has not been placed at the top of the column; and might we not ask at your hands that each person registering will be required to furnish to your office the date of his diploma?

Thanking you for past courtesies, and trusting we are not asking too much at your hands, we are,

Respectfully,

CHAS. T. GOENTNER, D.V.S., President,
Bryn Mawr, Pa.

WALTER L. HART, D.V.S., Secretary,
1411 North 4th Street, Philadelphia, Pa.

Philadelphia, Pa., January 9th, 1894.

HON. J. STERLING MORTON:—

HONORED SIR:—We, the officers and members of the Keystone Veterinary Medical Association, have read with very much pleasure and gratification your recommendation to the President, as conveyed to the people of the United States through his recent message, your desire to base all veterinary appointments in the Bureau of Animal Industry, over which you preside, upon the merit system, "and the wiping away of the infamous spoils system," which so often forces upon officers inefficient and incompetent men for these places, which are of so much importance. We also gratefully acknowledge your recommendation in regard to some method looking toward the extermination of "tuberculosis" from the milk cattle of our land, and we pledge you that in any way we may assist or serve you our association will only be too glad to render to you any aid within its power in carrying out these meritorious recommendations.

Respectfully yours,

CHAS. T. GOENTNER, D.V.S., President,
Bryn Mawr, Pa.

WALTER L. HART, D.V.S , Secretary,
1411 North 4th St., Phila., Pa.

The evening was well spent in discussing the subject of "Milk Fever," with a report from State Veterinarian Bridge of the recent outbreak of "tuberculosis" at Washington. Pa., and some remarks on the outbreak of "glanders" at Wilkesbarre, Pa., after which Dr. Hoskins reported a series of statistics in regard to varying weights of horses under injuries and various diseases, with the loss and gain measured from week to week. According to the record which he kept, horses with painful foot troubles lost most in weight, and much faster than any other disease. He also reported a case suffering with a spinal lesion of the lumbar region, which was emaciated, which gained an average of five pounds a day.

Dr. Chas. Lintz reported a case which he was called to look at suffering with colic, which was under the influence of some poisonous drug, which he, according to the symptoms the animal was laboring under, took for belladonna poison. Animal at the time looked at was staggering ; pulse hardly perceptible ; respiration, same ; pupil of the eye very much dilated ; mucous membrane very dry. Injected pilocarpine ; in fifteen or twenty minutes showed improvement, in a half hour respiration and pulse increased. Improved and lived four days and died from blood poison, from a large abscess which formed on the inferior part of the neck.

After some other business the meeting adjourned to meet second Tuesday in February, at which meeting Dr. Bridge will read a paper on "Glanders," and Dr. Lintz will read one on "Corns."

WALTER L. HART, *Secretary.*

---

